# Accuracy evaluation of 3D printed interim prosthesis fabrication using a CBCT scanning based digital model

**DOI:** 10.1371/journal.pone.0240508

**Published:** 2020-10-16

**Authors:** Young Hyun Kim, Bock-Young Jung, Sang-Sun Han, Chang-Woo Woo

**Affiliations:** 1 Department of Oral and Maxillofacial Radiology, Yonsei University College of Dentistry, Seoul, Korea; 2 Department of Advanced General Dentistry, Yonsei University College of Dentistry, Seoul, Korea; 3 Central Dental Laboratory, Dental Hospital of Yonsei University, Seoul, Korea; Virginia Commonwealth University, UNITED STATES

## Abstract

**Objectives:**

This study aimed to evaluate the marginal and internal gaps in 3D-printed interim crowns made from digital models of cone-beam computed tomography (CBCT) conversion data.

**Materials and methods:**

Sixteen polyvinylsiloxane impressions were taken from patients for single crown restorations and were scanned using CBCT. The scanning data were converted to positive Standard Triangulation Language (STL) files using custom-developed software. The fabricated stone models were scanned with an intraoral optical scanner (IOS) to compare the surface accuracy with the STL data obtained by CBCT. The converted STL files were utilized to fabricate interim crowns with a photopolymer using a digital light-processing 3D printer. The replica method was used to analyze the accuracy. The marginal and internal gaps in the replica specimen of each interim crown were measured with a digital microscope. The Friedman test and Mann-Whitney U test (Wilcoxon-signed rank test) were conducted to compare the measurements of the marginal and internal gaps with a 95% level of confidence.

**Results:**

The root-mean-square values of the CBCT and IOS ranged from 41.00 to 126.60 μm, and the mean was 60.12 μm. The mean values of the marginal, internal, and total gaps were 132.96 (±139.23) μm, 137.86 (±103.09) μm, and 135.68 (±120.30) μm, respectively. There were no statistically significant differences in the marginal or internal gaps between the mesiodistal and buccolingual surfaces, but the marginal area (132.96 μm) and occlusal area (255.88 μm) had significant mean differences.

**Conclusion:**

The marginal gap of the fabricated interim crowns based on CBCT STL data was within the acceptable range of clinical success. Through ongoing developments of high-resolution CBCT and the digital model conversion technique, CBCT might be an alternative method to acquire digital models for interim crown fabrication.

## Introduction

Interim crowns are an important component of the fabrication of permanent dental prostheses for several reasons: they protect pulpal and periodontal tissues, prevent teeth from micro-shifting, and maintain occlusal function [1, 2]. The conventional method of fabricating interim crowns is highly dependent on the operator’s skills, and allergic contact stomatitis can be caused by residual monomers [3, 4]. Additionally, voids generated during the material mixing procedure might weaken the mechanical strength of a manually manufactured interim crown, causing it to fracture [5].

With the advancement of digital dentistry, various computer-aided design and computer-aided manufacturing (CAD/CAM) methods and 3-dimensional (3D) printing methods have been widely applied in the dental field [6–9]. Based on previous studies, fabricating interim crowns with the CAD/CAM systems can solve some of the shortcomings of conventional methods [5, 10–12]. Moreover, some researchers have found that interim crowns fabricated with 3D printing have outstanding marginal and internal fits, and were more accurate than interim crowns fabricated with the conventional method or even with the CAD/CAM milling method [9, 13].

Obtaining an accurate 3D digital model is essential to begin the digital prosthetic process [14]. A digital model can be acquired by various scanning methods, such as an intraoral scanner (IOS), extraoral scanner (EOS), or cone-beam computed tomography (CBCT) [14–16]. IOS can directly capture the oral cavity and provide data with clinically applicable precision [17]. However, in most studies that investigated the relationship between IOS and the scanning range, scan errors became more serious as the size of the scan area increased [18–20]. In addition, IOS has a learning curve that affects the image quality and makes the device difficult to use during the initial period [21, 22]. EOS is another well-known method of obtaining digital models, but it involves the inconvenient step of creating a stone model [23]. Furthermore, an important limitation of both optical scanning methods is the loss of information in areas where light from the source cannot reach, such as between adjacent teeth or in an undercut [24–26].

CBCT, which provides essential 3D diagnostic information to dentists [27], has recently been considered as an alternative scanning method to acquire digital models for use in orthodontics, surgical guides, and restorative prostheses [28–31]. It has been verified that the accuracy of CBCT-scanned data is within the clinically acceptable range, although CBCT-scanned data are less accurate than those obtained by optical scanning [14, 28].

Currently, the latest developments in some high-performance CBCT devices allow clinicians to obtain data with small voxel sizes ranging between 75 and 100 μm [14, 32]. CBCT data in the Digital Imaging and Communications in Medicine (DICOM) format must be converted to the Standard Triangulation Language (STL) format in order to fabricate 3D-printed prostheses [28]. Both voxel size and the capabilities of conversion software affect the accuracy of STL files converted from CBCT-acquired data [14, 28, 29]. However, no trials have evaluated the accuracy of 3D-printed interim crowns using the latest CBCT devices. Thus, this study aimed to introduce a special image acquisition mode of CBCT and a newly developed conversion software, and to evaluate the usability of 3D-printed interim crowns made from digital models of CBCT data.

## Materials and methods

**Fig 1:**
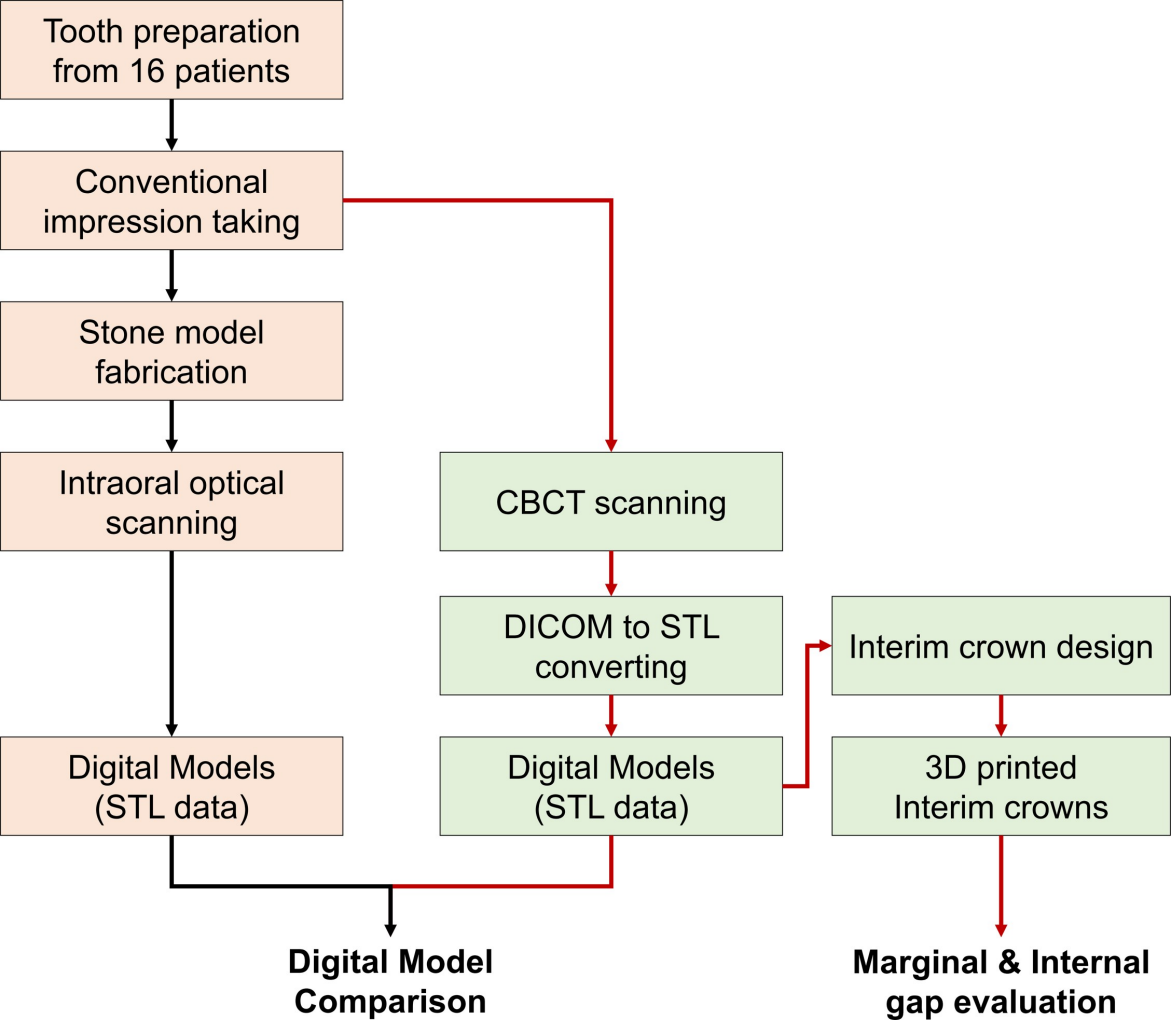
The overall experimental workflow in this study. Two devices were used in this experimental study to obtain digital models: an intraoral scanner (black line) and CBCT (red line).

Fig 1 presents the experimental workflow of our study.

### 1. Subjects

Sixteen patients who visited Yonsei University Dental Hospital between January and April 2017 for single fixed dental prostheses were selected. The following inclusion criteria were applied: no tooth loss in the maxillary and mandibular dentition, no orthodontic appliances, and no crowding. The Institutional Review Board of Yonsei Dental Hospital reviewed and approved this study (IRB No. 2-2016-0048). All participants were adults and wrote the informed consent with a guaranteed opportunity for refusal prior to the research proceeded.

### 2. Conventional impression procedure and CBCT scanning

**Fig 2:**
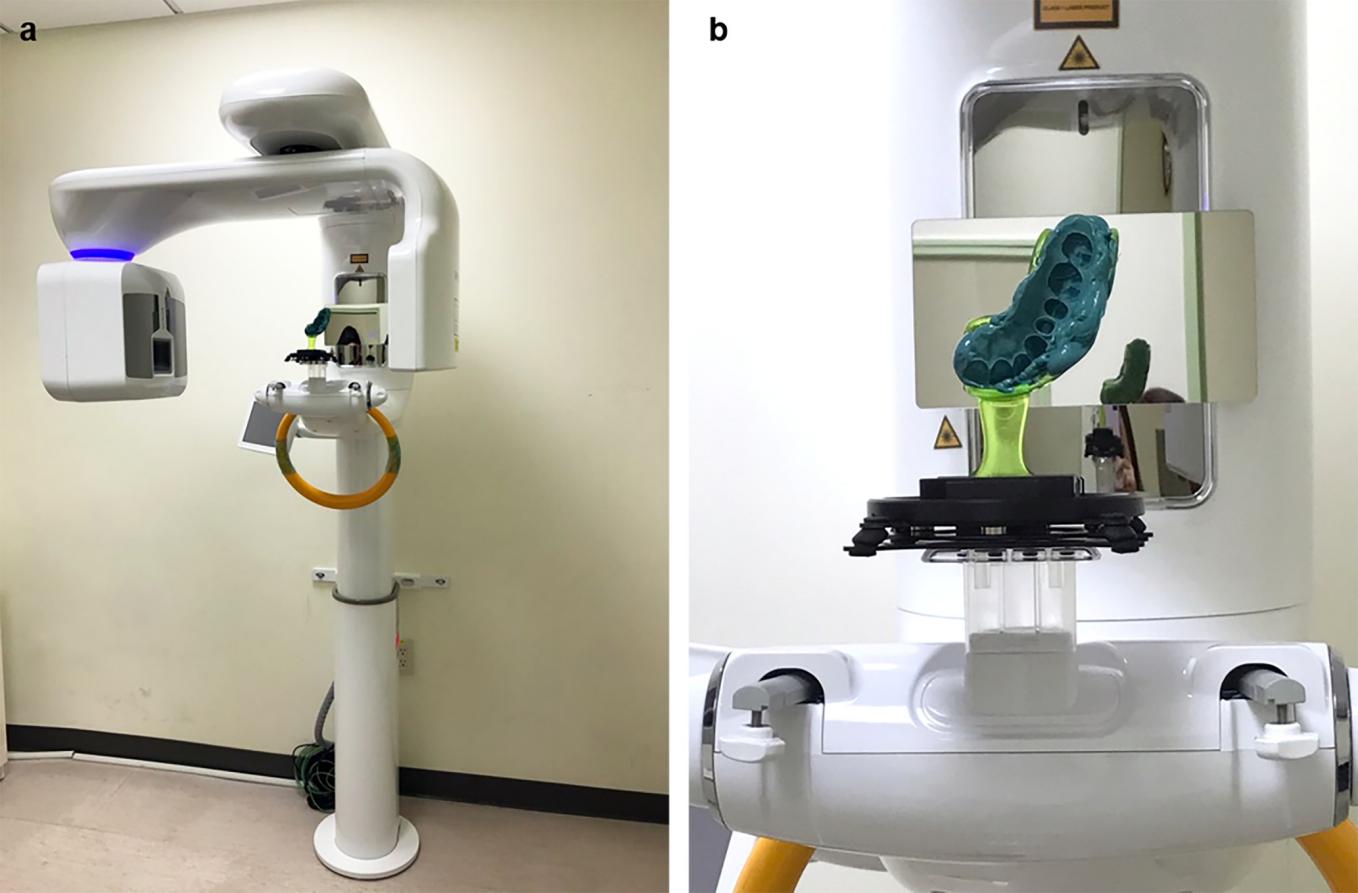
Cone-beam computed tomography (CBCT) scanning. (a) CBCT apparatus (RAYSCAN α+) and (b) a polyvinylsiloxane impression fixed on a customized chin rest provided by the manufacturer for scanning.

A skilled prosthodontist prepared a single tooth for a gold crown using round-tipped, tapered diamond burs and sufficient water coolant from high and low-speed friction angles, ensuring both the minimum recommended clearance (1 mm for nonfunctional cusps and 1.5 mm for functional cusps) and the chamfer configuration margin. A conventional impression was made using a polyvinylsiloxane (PVS) elastomer combining a low viscosity for injection and a high viscosity for putty in a stock tray (3M EPSE Monophase; 3M Deutschland GmbH, Neuss, Germany). After the PVS impression was taken, a provisional restoration made of a self-curing acrylic resin (Jet Tooth Shade, Wheeling, IL, USA) was delivered to the patient's prepared tooth chairside. A customized chin rest made by the manufacturer was used to fix the PVS impression on the CBCT apparatus for scanning (RAYSCAN α+; Ray Co., Ltd., Hwaseong-si, Korea) (Fig 2A and 2B). A skilled radiographic technician performed CBCT scanning with an object scanning mode of 90 kVp and 6 mA, a voxel size of 100 μm, a field of view of 90×50 mm^2^, and a duration of 14 seconds. After CBCT scanning, high-strength dental stone was poured in the PVS impression to fabricate a stone model (MG Crystal Rock; Maruishi Gypsum Co., ltd, Osaka, Japan).

### 3. Development of customized conversion software

**Fig 3:**
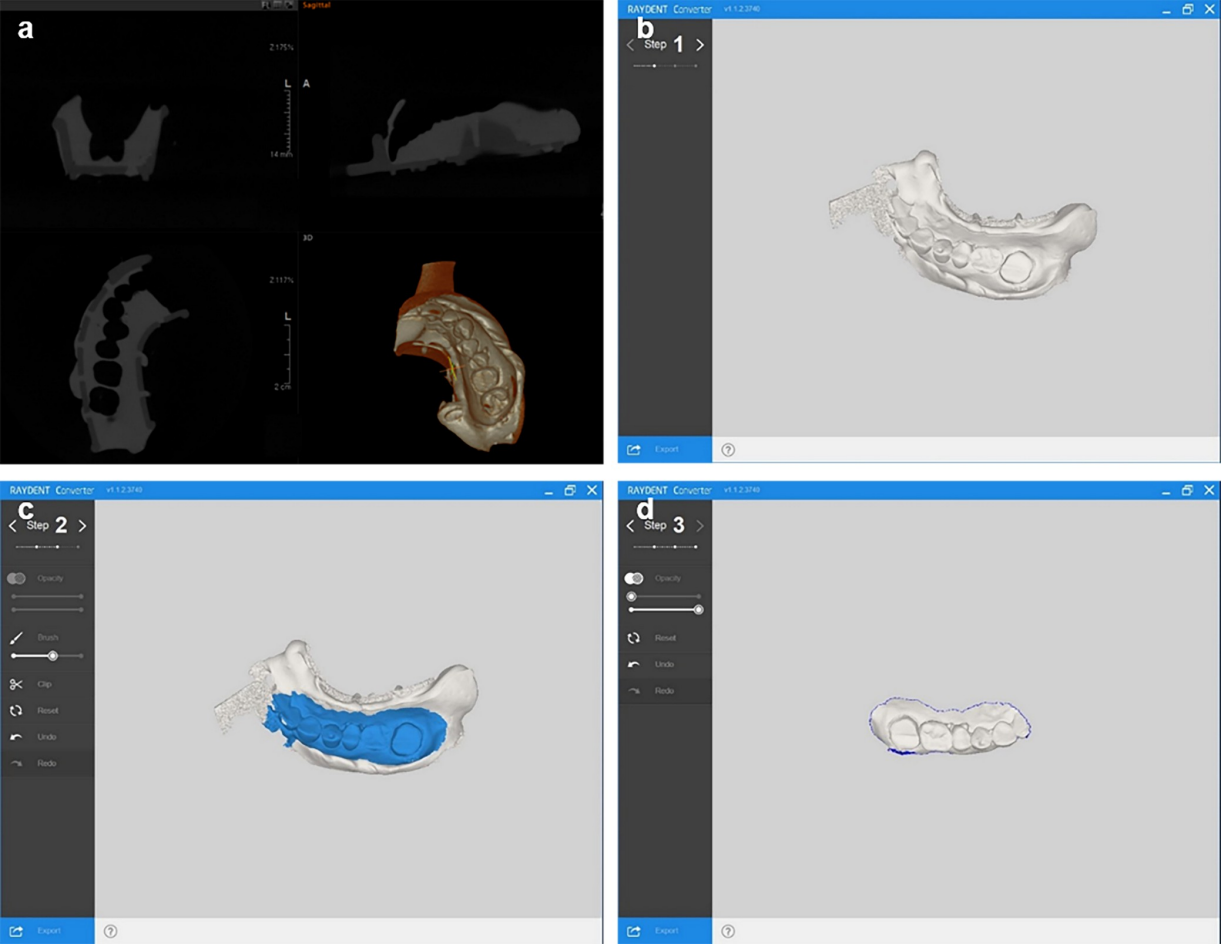
Conversion from the DICOM format to STL files using customized conversion software. (a) DICOM file loading, (b) negative data, (c) selection of the positive range, and (d) conversion to a positive format.

**Fig 4:**
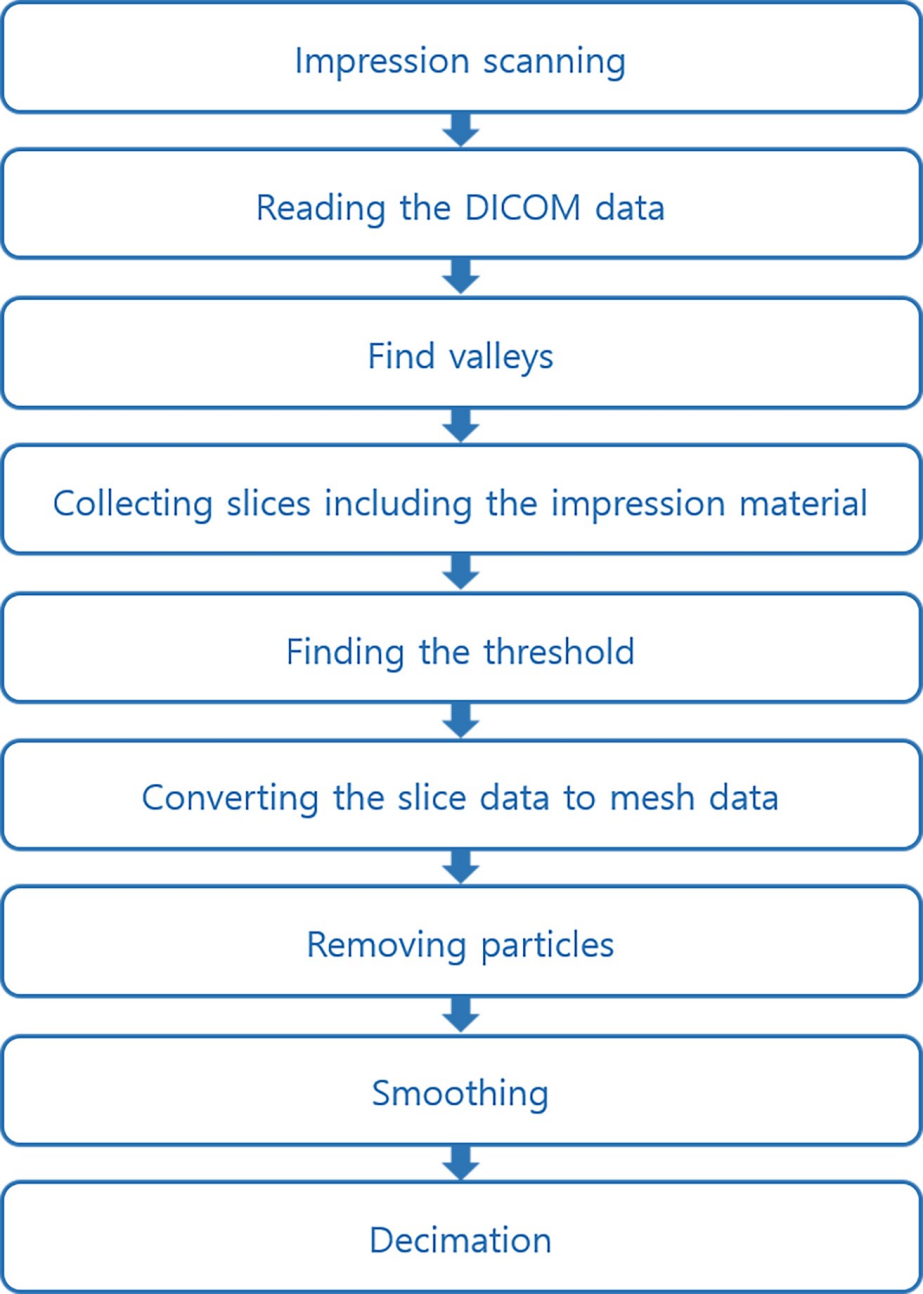
Flow chart of the conversion process.

**Fig 5:**
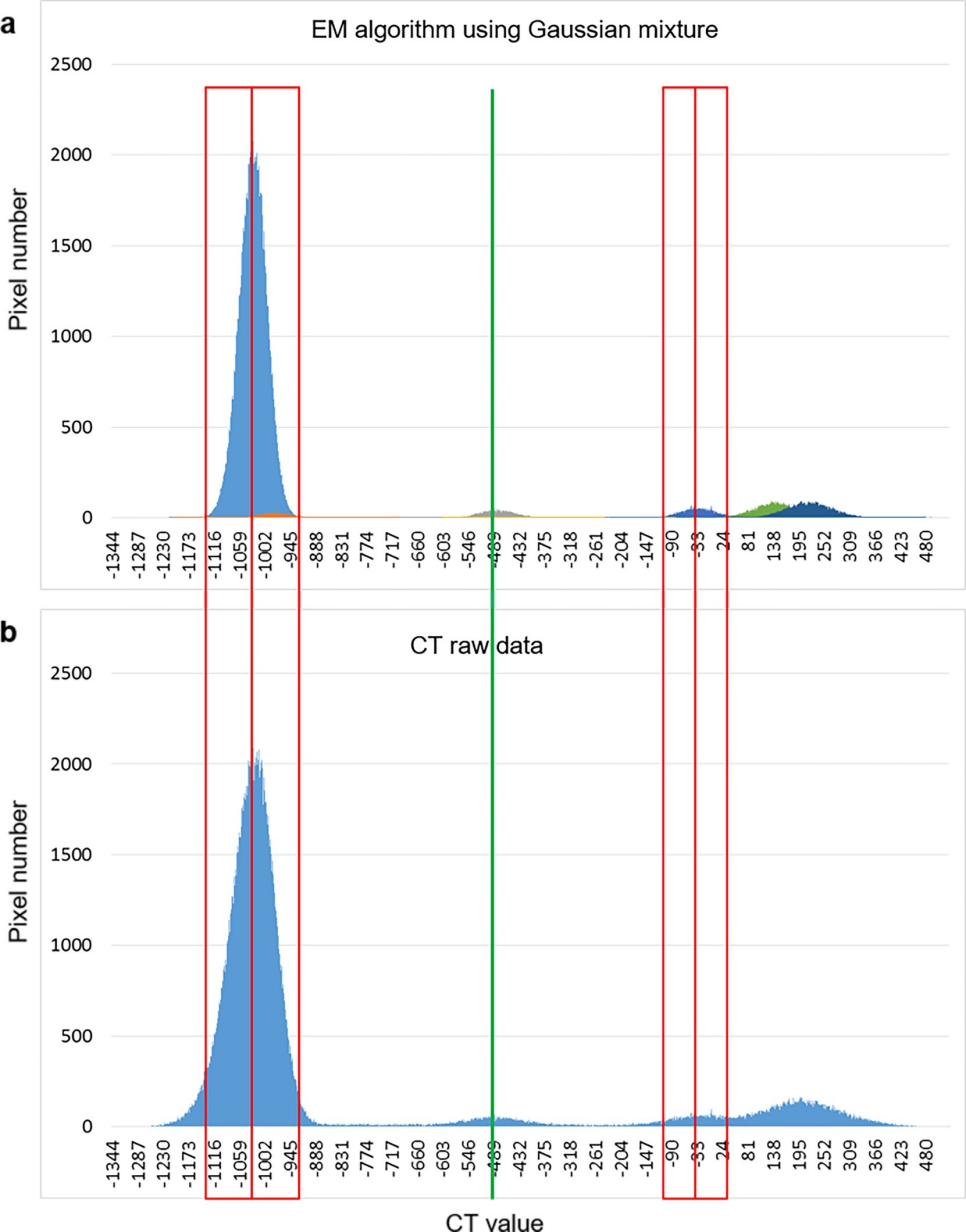
Expectation-maximization (EM) algorithm using Gaussian mixtures for auto-thresholding of the polyvinylsiloxane (PVS) impression. (a) The threshold of PVS impressions found using the EM algorithm, (b) the raw data of computed tomography values before using the EM algorithm.

Customized conversion software (RAYDENT Converter ver.1.1.2; Ray Co., Ltd., Hwaseong-si, Korea) developed for acquiring accurate conversion digital data (STL data) from the PVS impression (Fig 3A–3D). The flowchart of the overall conversion process is shown in Fig 4. The software utilized histogram-based valley estimations and an expectation-maximization (EM) algorithm to perform optimized auto-thresholding of PVS segmentation (Fig 5A and 5B) [33, 34]. The estimated parameters that the EM algorithm assumed were a histogram-based valley and the number of segmentation clusters [33]. The EM algorithm searched for an optimal threshold based on the assumption that the classes of the object and background follow a general Gaussian distribution [34]. The medium-body PVS impression was scanned with CBCT to establish a calibrated value to pick up the exact valley of the PVS impression after the EM algorithm was applied. Thus, detecting the valley of the impression material designated an area of conversion, which enhanced the efficiency, and the border of the impression material was then calculated to find the threshold accurately. Generally, the conversion process of mesh smoothing results in a loss of data. To minimize this source of error, Taubin’s fair surface design algorithm was used to compensate for the loss of data by using the surface manipulation technique [35]. By using this automated algorithm, positive STL data for the impression material can be obtained with minimal conversion errors.

### 4. Intraoral scans of stone models

**Fig 6:**
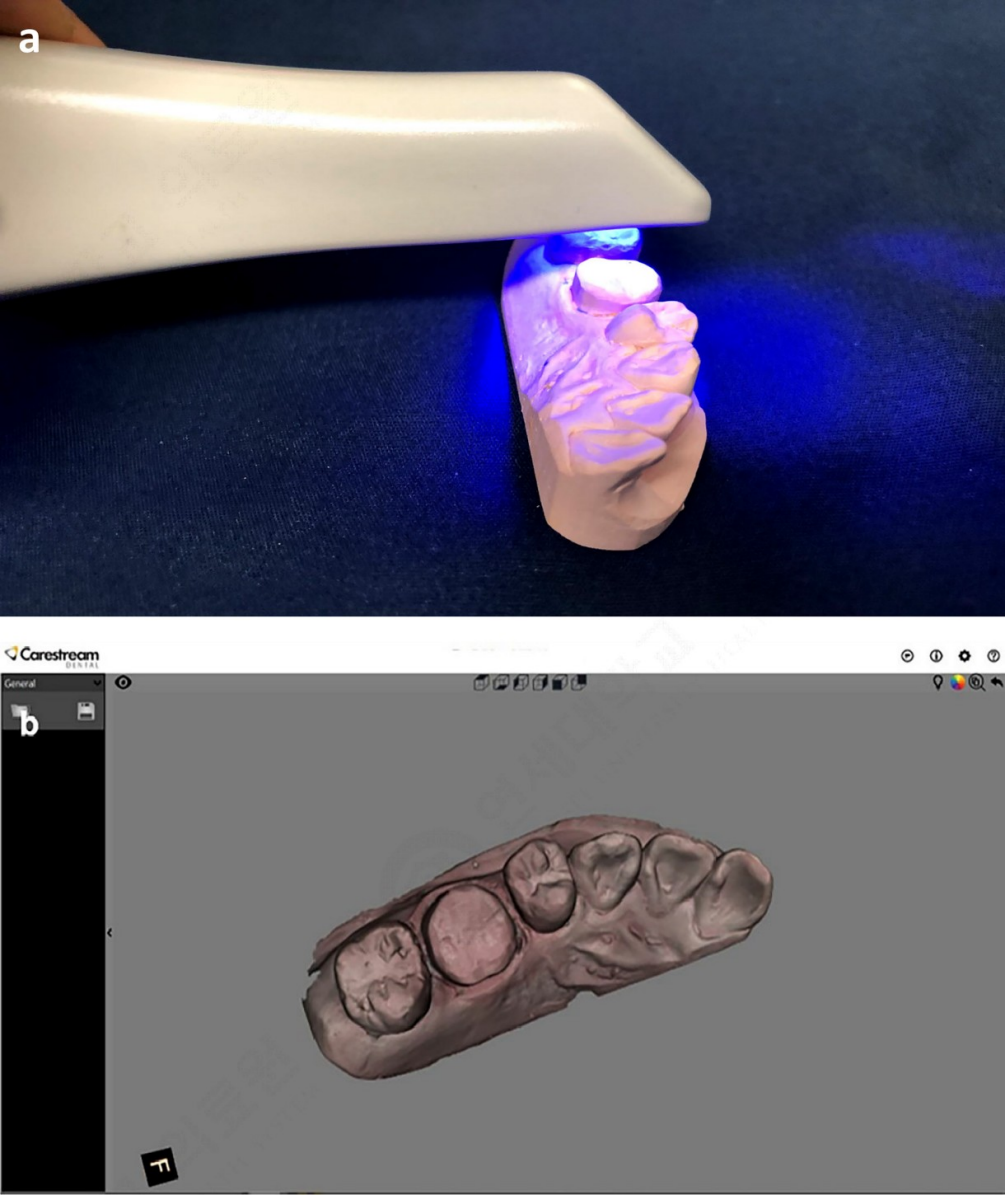
Intraoral optical scanning. (a) Scanned with an intraoral optical scanner (CS 3600), (b) saved in the STL format.

**Fig 7:**
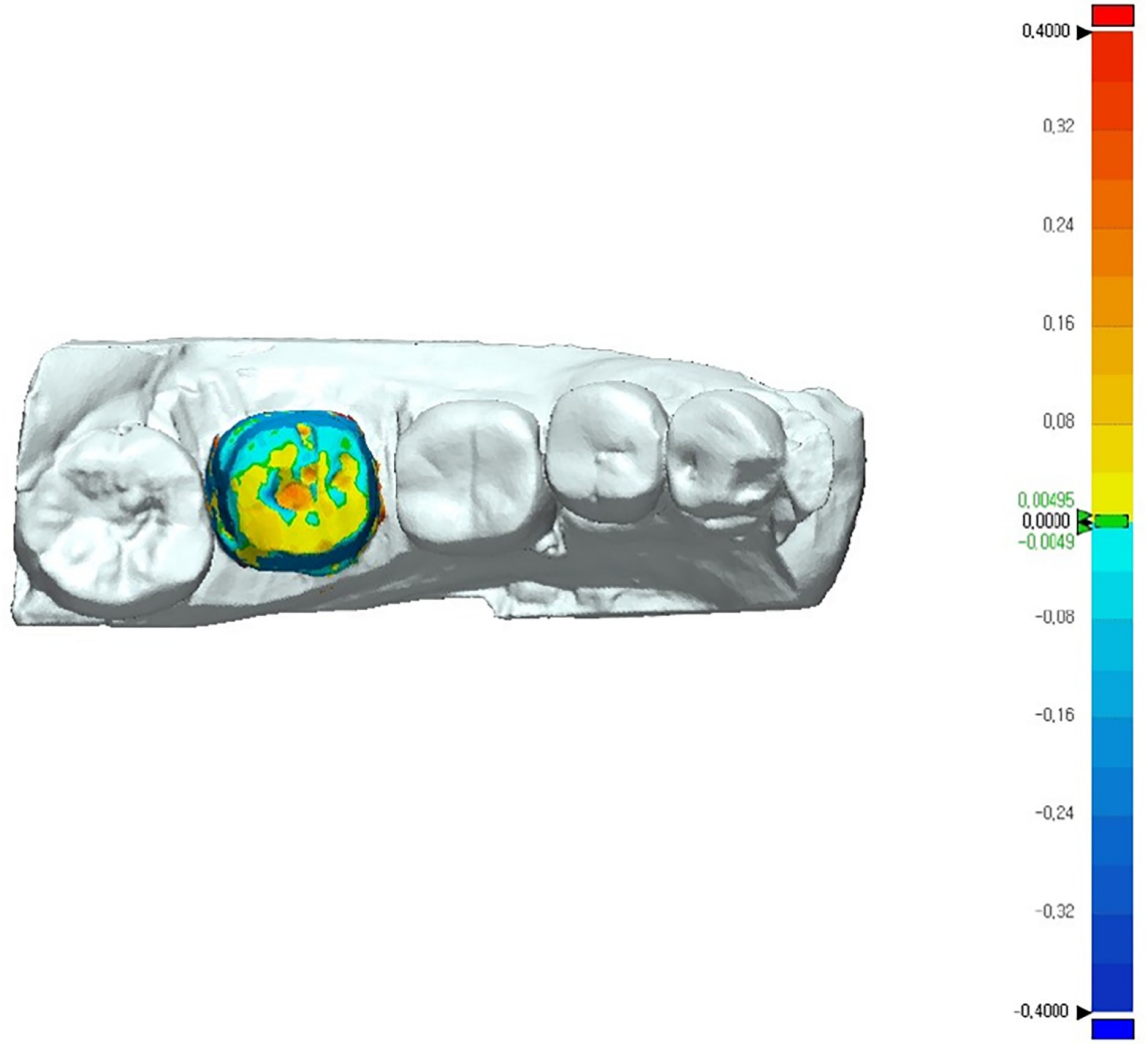
Three-dimensional deviations between the comparison and reference STL files using GeoMagic software.

Digital models were obtained from the stone models using an IOS (CS 3600; Carestream, Rochester, NY, USA) and saved in the STL format (Fig 6A and 6B), and the IOS-scanned digital model was utilized as reference data. The surface accuracy of the digital model based on the CBCT scans was compared with the reference data using GeoMagic software (GeoMagic Control X; Braunschweig, Germany). The auto-segmentation function was utilized to separate the prepared area, which was regarded as the true area in the superimposition of the 2 sets of data, from the other segments. In order to reduce the manual error in superimposing the 2 sets of data and to ensure reliability, the ‘initial alignment’ and ‘best fit’ functions were used, and the software was set to automatically recognize the figures to improve its accuracy. The 3D deviations of the prepared tooth area between the comparison and reference STL data were calculated by measurements made using a color-coded scale based on root-mean-square (RMS) values (Fig 7).

### 5. Design of the interim crowns and 3D printing

**Fig 8:**
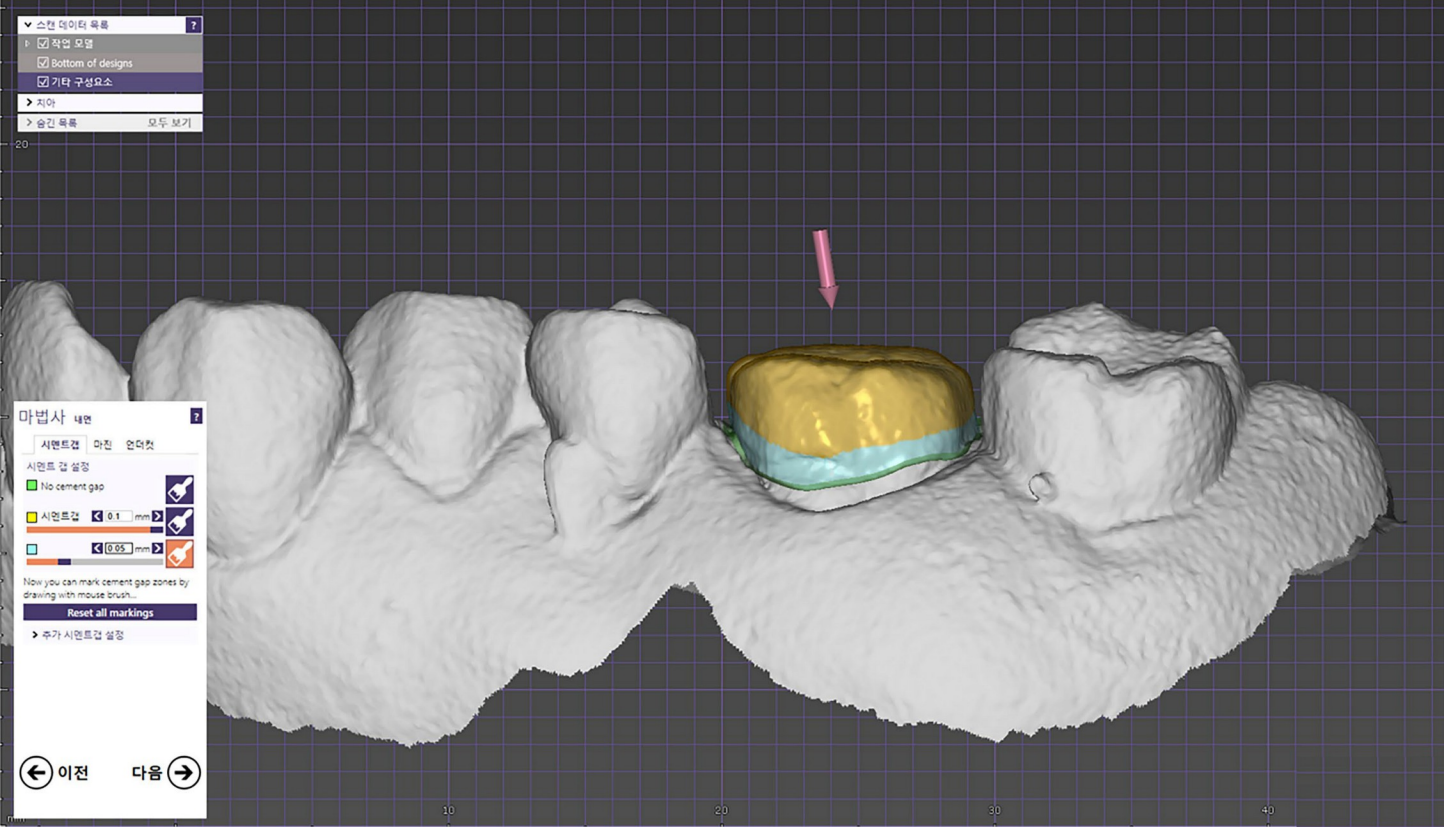
Computer-aided design for the interim crown.

**Fig 9:**
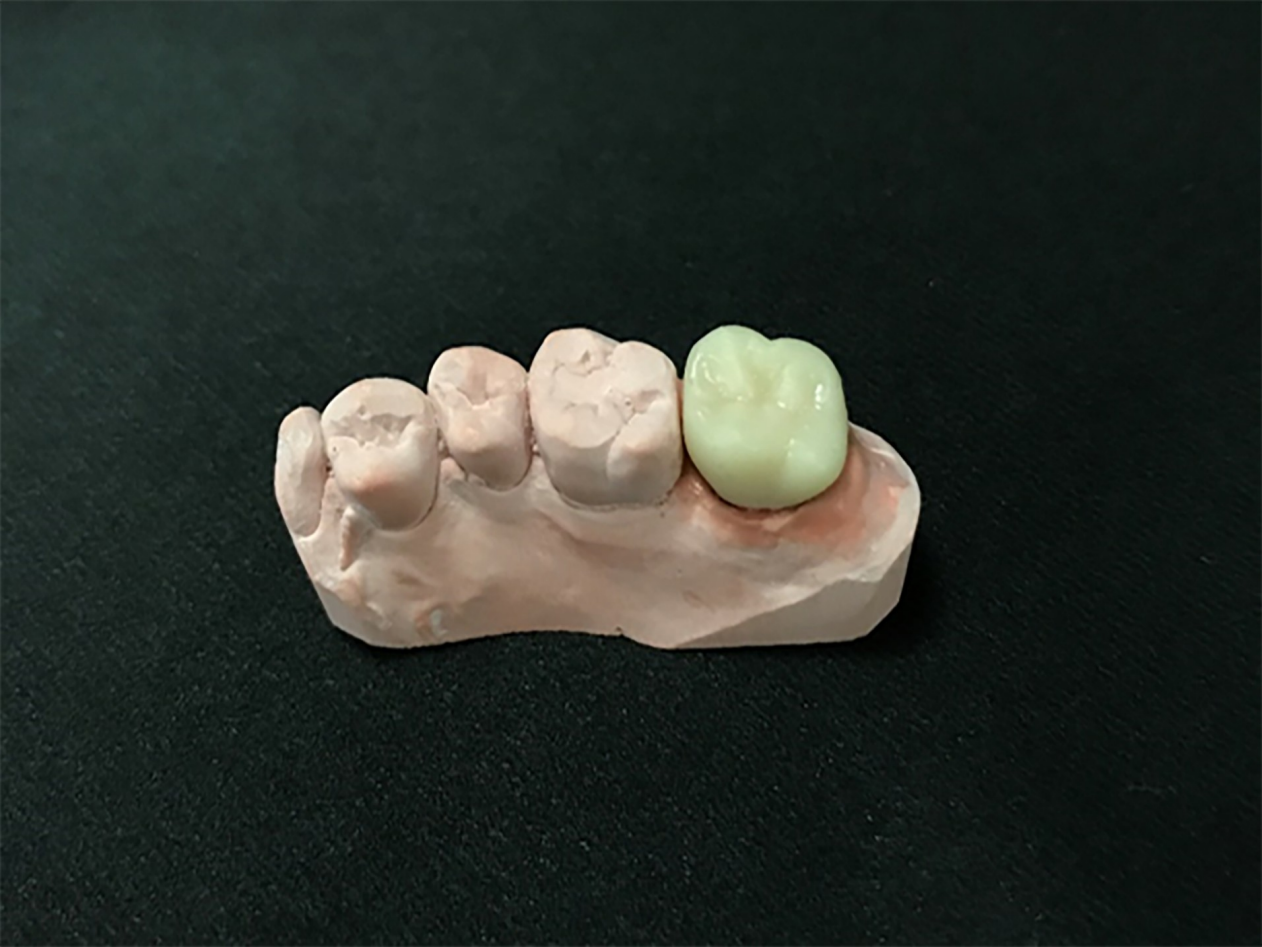
Fabricated interim crown with a photopolymer using a digital light-processing 3D printer.

A skilled dental technician with 10 years of experience designed the interim crowns based on the CBCT digital models using CAD software (Exocad GmbH, Darmstadt, Germany). In accordance with Mai et al. [13], the cementing space was set at 50 μm near the margins of the crowns and 100 μm for the rest of the internal space (Fig 8). The interim crowns were printed with a digital light-processing 3D printer (RAYDENT Studio; Ray Co., Ltd., Hwaseong-si, Korea) using a photopolymer material (RAYDent C&B; Ray Co., Ltd., Hwaseong-si, Korea) (Fig 9).

### 6. Measurement of the marginal gap and internal gap

**Fig 10:**
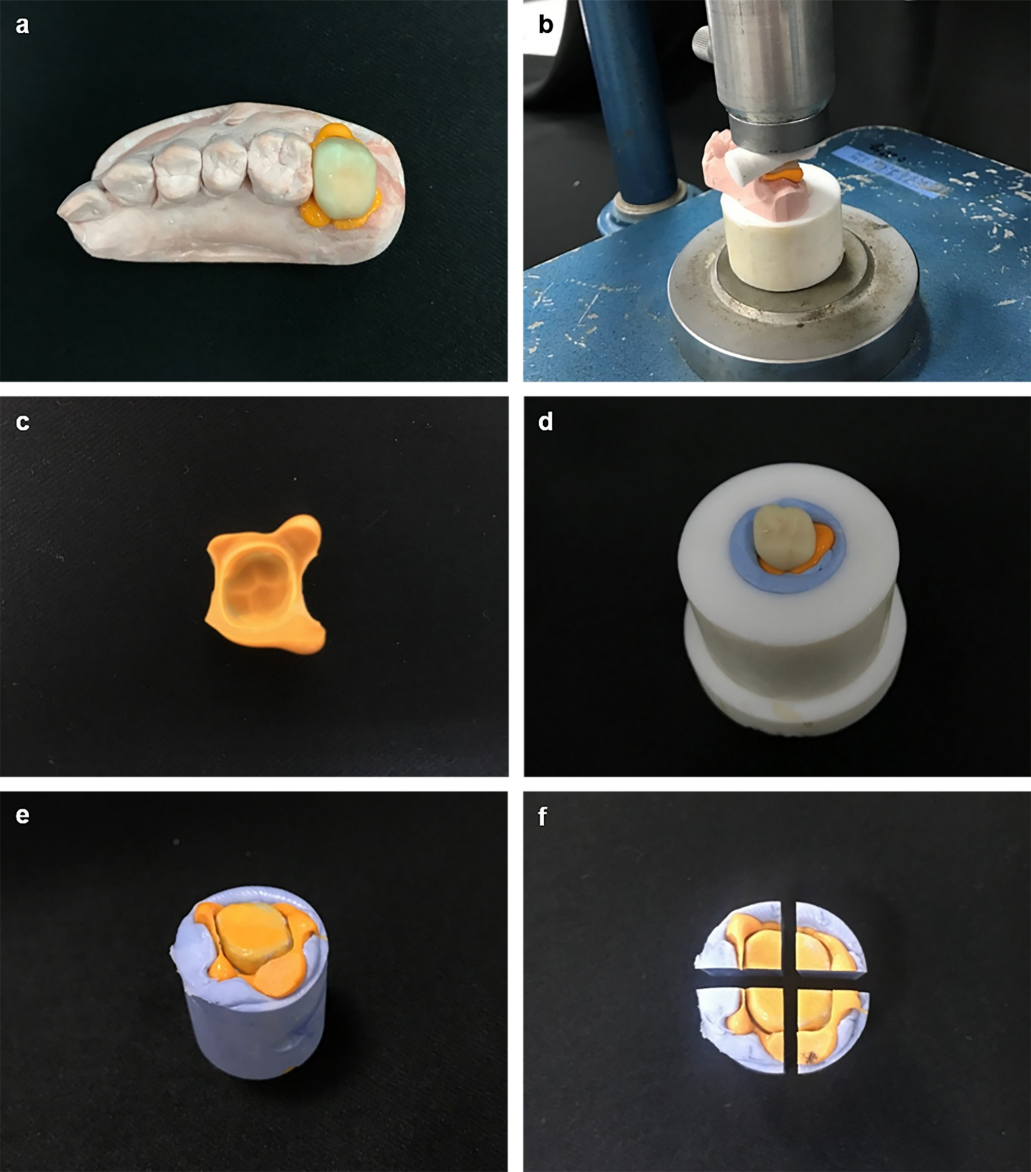
Two-dimensional replica method. (a) Filled with light-body impression materials and seated together with the stone models, (b) 50 N of pressure was applied for 5 minutes using static load equipment, (c) the pressure was removed from the stone models after polymerization, (d) cylinder-shaped molds were filled with putty, (e) the specimen was created, (f) the specimen was sectioned buccolingually and mesiodistally at the midline.

The replica technique [36] was used to measure the accuracy of the 16 interim crowns. This technique involves measuring the thickness of the cross-section of the silicone replica obtained after filling the gap between the crown and prepped tooth with silicone material [37]. The manufactured interim crowns were filled with light-body polyvinylsiloxane impression materials (Aquasil Ultra XLV, Dentsply DeTrey GmbH, Konstanz, Germany) and seated on the stone die of the prepared tooth (Fig 10A). A pressure of 50 N was applied on the interim crown for 5 minutes [38], using static load equipment. Cotton rolls were placed onto every specimen to deliver a consistent pressure across the areas (Fig 10B).

**Fig 11:**
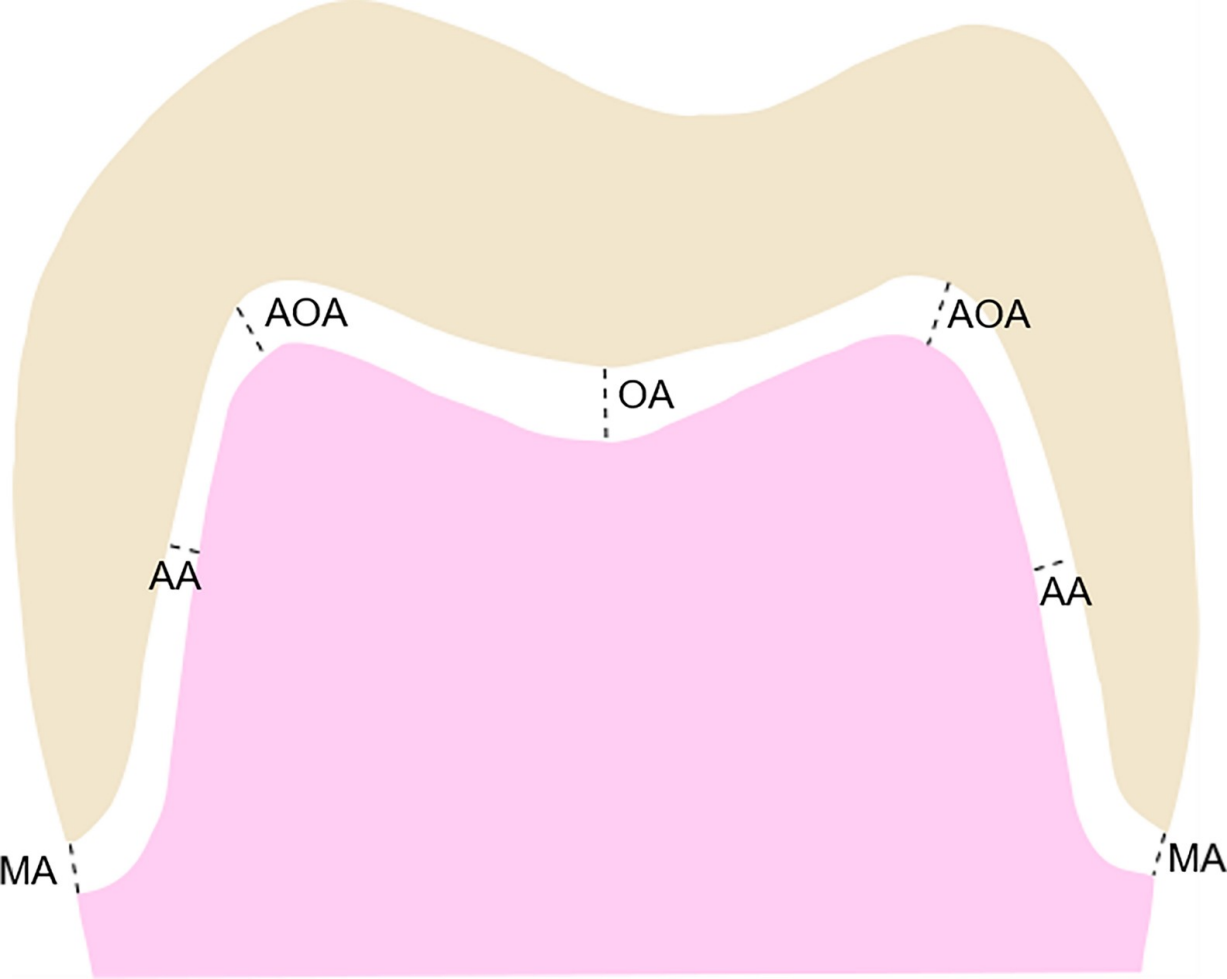
Schematic representation of the measurement points in a cross-section of a specimen. MA: marginal area; AA: axial area; AOA: axio-occlusal angle; OA: occlusal area.

**Fig 12:**
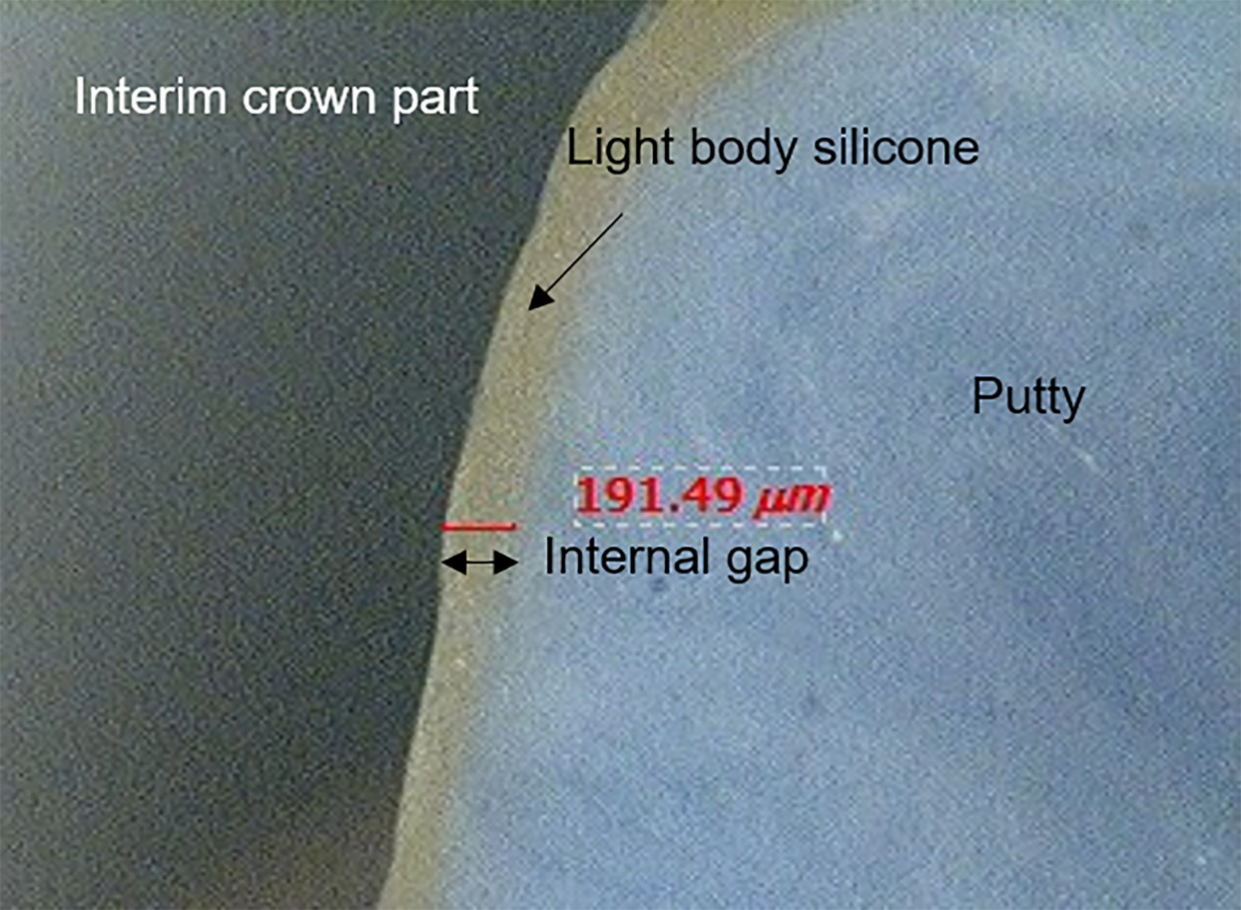
Measurement of marginal and internal gaps using a digital microscope (magnification ×100).

Cylinder-shaped molds filled with putty impression materials (Aquasil Soft Putty; Dentsply DeTrey GmbH, Konstanz, Germany) were used to create the specimens and to support the shape (Fig 9C and 9D). The replica specimens were removed from the mold after polymerization of the putty, and each replica specimen was sectioned mesiodistally and buccolingually at the midline (Fig 10E and 10F). The thickness of the light-body impression material was measured in 4 directions using mesiodistal and buccolingual sections. Four measurement points were examined: (1) the marginal area (MA), (2) the axial area (AA), (3) the axio-occlusal angle (AOA), and (4) the occlusal area (OA) (Fig 11). The marginal and internal gaps of each specimen were measured with ×100 magnification using digital microscope system (Hirox KH-1000 Hi-scope 3D Power, Hirox-USA, Inc., Hackensack, NJ, USA) (Fig 12).

### 7. Statistical analysis

Statistical analyses of the marginal and internal gaps were performed and the means, standard deviations, medians, and interquartile ranges of the measurements were calculated. The normality assessment did not support the presence of a normal distribution (*P* < 0.01). The Mann-Whitney U test was conducted to determine whether the measurement points of the marginal and internal gaps were significantly different between the mesiodistal and buccolingual surfaces. The Friedman test and Wilcoxon-signed rank test were performed to compare the mean discrepancies among the measured points of the interim crowns. Statistical analyses were performed using SPSS Statistics for Windows, version 23.0 (IBM Corp., Armonk, NY, USA) with a 95% level of confidence.

## Results

The RMS values of the IOS-scanned and CBCT-scanned digital models ranged from 41.00 to 126.60 μm; the mean was 60.12 (± 21.35) μm (Table 1).

**Table 1:** The root-mean-square values of the IOS-scanned and CBCT-scanned digital models (μm).

Measurements	RMS Mean	RMS SD	RMS Min	RMS Max
Values	60.12	21.35	41.00	126.60

SD: standard deviation; Min: minimum; Max: maximum.

**Fig 13:**
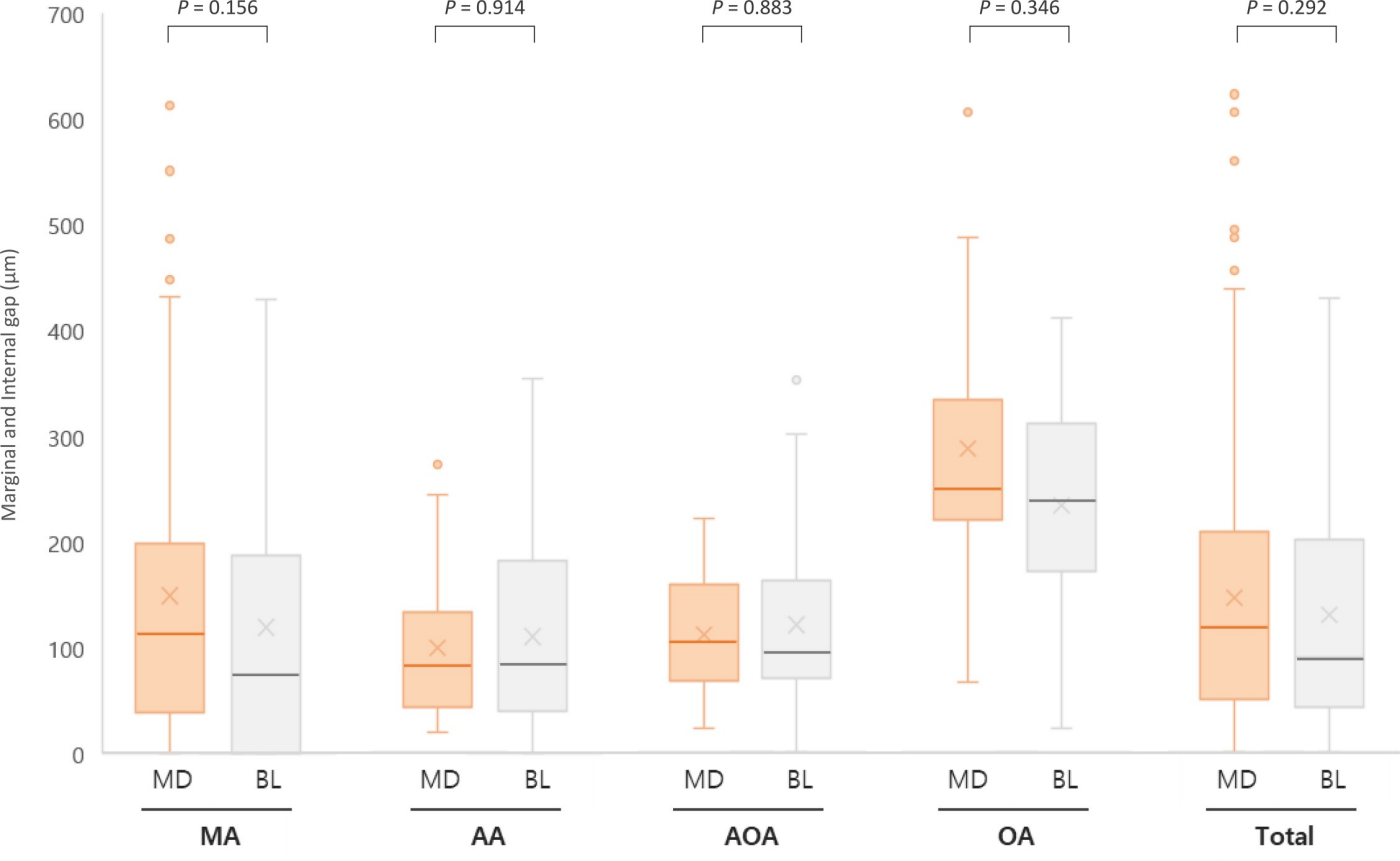
Comparison of the marginal gap and internal gap between mesiodistal and buccolingual surfaces. MD: mesiodistal; BL: buccolingual; MA: marginal area; AA: axial area; AOA: axio-occlusal angle; OA: occlusal area. P-values were obtained using the Mann-Whitney U test.

**Fig 14:**
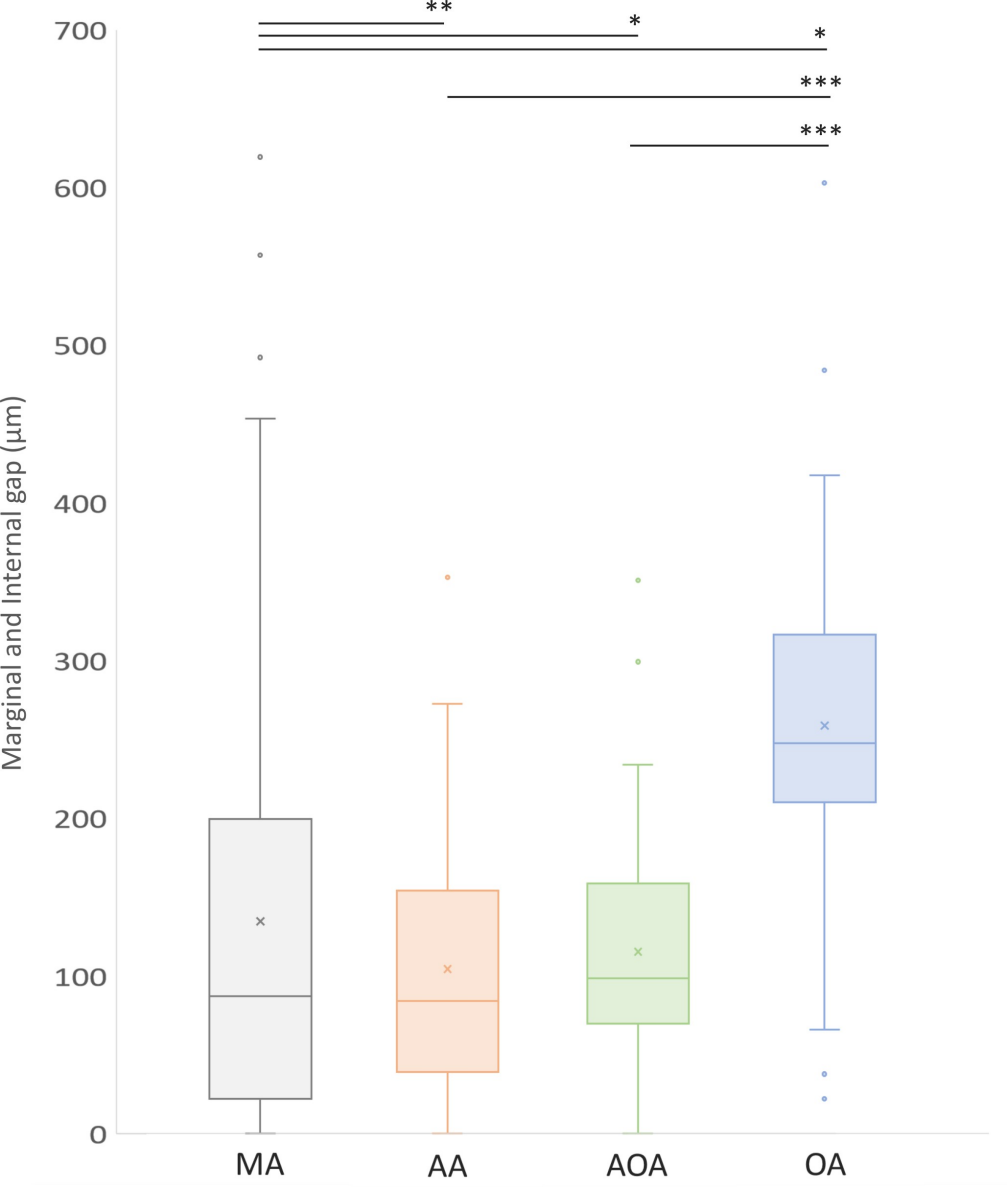
Comparison of the marginal and internal gap according to the measurement area. MA: marginal area; AA: axial area; AOA: axio-occlusal angle; OA: occlusal area. * p<0.05; ** p<0.01; *** p<0.001 P-values were obtained using the Mann-Whitney U test.

The descriptive results of the measured gap are summarized in Table 2. The mean values of the marginal, internal, and total gaps were 132.96 (± 139.23) μm, 137.86 (± 103.09) μm, and 135.68 (± 120.30) μm, respectively. As shown in Fig 13, no marginal and internal gap between the mesiodistal and buccolingual surfaces showed a statistically significant difference in any measurement area. The overall mean (average of the marginal and internal gap) and standard deviation values for all measurement points at the mesiodistal and buccolingual surfaces were 143.51 (± 127.51) μm and 127.86 (± 112.52) μm, respectively; no statistically significant differences were found. When a comparison of mean discrepancies was conducted, the marginal and occlusal area had significant mean differences compared to those the other measured areas, with 95% significance (MA mean: 132.96 μm, median: 86.00 μm, interquartile range: 21.74 to 197.20 μm; OA mean: 255.88 μm, median: 244.94 μm, interquartile range: 207.57 to 312.74 μm) (Fig 14). There was no significant discrepancy between the AA and AOA (*P* = .150).

**Table 2:** Descriptive statistical analysis of marginal and internal gaps (μm).

Measurements	Samples	Mean	SD	Median	Interquartile Range
Marginal gap	64	132.96	139.23	86.00	177.61
Internal gap	160	137.86	103.09	109.82	143.11
Total gap	224	135.69	120.30	108.70	156.94

SD: standard deviation.

## Discussion

In applications of CAD/CAM in the dental field, oral digital models have primarily been used directly, through intraoral scans, or indirectly, through extraoral optical scans of conventional stone models [39]. A recently developed IOS system can reduce patients’ discomfort through its smaller head tip size, whereas scan errors and scanning time increase as more images being captured continuously [14, 40]. Although the indirect scanning of model casts produces higher-resolution images [39], it can produce incomplete intraoral information due to limited light exposure in the interproximal spaces and the possibility of distortion caused by handling the stone model [24]. A CBCT digital model eliminates the process of fabricating a cast model and acquires information in areas such as undercuts and adjacent teeth without influence of the scanning light angle.

Based on the findings of previous studies, digital models acquired by IOS are considered to have clinically acceptable accuracy [41]. In this study, we superimposed digital models based on CBCT data from the PVS impressions with those of the IOS data from stone models to evaluate surface discrepancies. The RMS values of the two digital models ranged from 41 to 126 μm, with an average of 60 μm. These results are within the range of 40 to 200 μm that has been reported as a clinically acceptable discrepancy between stone models and digital models [42–44].

The accuracy of the marginal fit is considered to be one of the most important criteria for evaluating the quality of dental prostheses. According to previous studies, the average discrepancy of marginal fit has been reported to range from 177 μm to 400 μm for interim crowns [45, 46], and from 100 μm to 200 μm for definitive prostheses [47–50]. The marginal gap of the interim crown fabricated from a CBCT-based digital model in this study was measured to be 132.96 μm. Given that internal and marginal fits are generally less crucial for an interim crown than for a definitive crown, the results of our experiment are within the clinically acceptable range.

The marginal gaps between the mesiodistal and buccolingual surfaces of the CBCT-digitalized, 3D-printed interim crowns did not show statistical significance in any measurement area, whereas there were significant differences between the measured discrepancies depending on measurement points (e.g., MA, AA, AOA, and OA). Kim et al. [14] reported that a study assessing linear length values of the CBCT-scanned digital models with the values acquired from a coordinate measuring machine resulted in greater errors in the Z-axis (height) direction than in the X- and Y-axis directions. Moreover, in a previous study that evaluated the internal gap of 3D-printed interim crowns fabricated by the silicone replica method, the discrepancy of the occlusal area was found to be larger than those of other areas. In this experiment, the occlusal area had the highest internal gap, with an average of 255.88 μm (median: 244.94 μm), which was a higher error value than the average of the marginal gap (mean: 132.96 μm, median: 86.00 μm). It can be assumed that errors may accumulate during the processes of conventional impression taking, CBCT scanning and 3D printing. Despite the large internal gaps of the 3D-printed crowns, the measured errors are still within the clinically permitted range of interim crowns (177–400 μm) [45, 46].

Meanwhile, the interquartile range of MA was between 21.74 μm and 197.20 μm, showing the largest variation among the values acquired in the other measurement areas. Additionally, it can be seen that the range of the marginal and internal gaps were wide regardless of the measurement points. Some characteristics of CBCT devices, such as the cone-shaped beam and X-ray scattering, affect DICOM data, resulting in a higher inconsistency of grayscale values and worsened low-contrast resolution [32]. The lower scanning resolution of CBCT than that of IOS may be another factor in the high discrepancy between these values. It would be better to use CBCT devices, which provides a high resolution of 100 μm or less, to obtain more accurate marginal edge information on prepared teeth.

Apart from the resolution of CBCT, the capabilities of the conversion software can also affect the marginal and internal fits of the interim crown. Although a wide range of conversion software, including open-source and commercial software, has been developed, insufficient information exists regarding the capabilities of conversion software. Szymor et al. [51] reported that the accuracy of segmentation is the primary factor that effects the overall precision of printed models. To obtain a more accurate digital model, we developed customized conversion software adapting specific algorithms. Since the grayscale values depend on the impression materials, a calibration process after the CBCT scan will help to obtain an accurate STL file.

Many researches have been conducted by taking impressions in phantoms. This study was performed by data obtained from the oral cavity of a real patient, and it was revealed that the interim crowns fabricated in this way were suitable. It is a necessary process before clinical use. However, this study has a limitation that the margin gap of the fabricated interim crown was checked on the stone model rather than in patient’s oral cavity. When evaluating the 3D printing of the final prosthesis in the future, the accuracy evaluation methods should be supplemented.

In conclusion, the marginal gap of the 3D-printed interim crowns that were designed using CBCT-acquired digital models was within the acceptable range for clinical success. Through ongoing development of high-resolution CBCT and conversion software, CBCT could be widely used for digital model acquisition and prosthesis fabrication.
